# The cardiovascular safety of incretin-based therapies: a review of the evidence

**DOI:** 10.1186/1475-2840-12-130

**Published:** 2013-09-06

**Authors:** John R Petrie

**Affiliations:** 1 Institute of Cardiovascular and Medical Sciences, University of Glasgow, 126 University Place, Glasgow G12 8TA, UK

**Keywords:** Type 2 diabetes mellitus, Cardiovascular safety, Incretin, GLP-1, GLP-1 receptor agonists, DPP-4 inhibitors

## Abstract

Cardiovascular disease (CVD) is a leading cause of morbidity and mortality in people with diabetes and therefore managing cardiovascular (CV) risk is a critical component of diabetes care. As incretin-based therapies are effective recent additions to the glucose-lowering treatment armamentarium for type 2 diabetes mellitus (T2D), understanding their CV safety profiles is of great importance. Glucagon-like peptide-1 (GLP-1) receptor agonists have been associated with beneficial effects on CV risk factors, including weight, blood pressure and lipid profiles. Encouragingly, mechanistic studies in preclinical models and in patients with acute coronary syndrome suggest a potential cardioprotective effect of native GLP-1 or GLP-1 receptor agonists following ischaemia. Moreover, meta-analyses of phase 3 development programme data indicate no increased risk of major adverse cardiovascular events (MACE) with incretin-based therapies. Large randomized controlled trials designed to evaluate long-term CV outcomes with incretin-based therapies in individuals with T2D are now in progress, with the first two reporting as this article went to press.

## Introduction

Cardiovascular disease (CVD) is a leading cause of morbidity and mortality in people with diabetes, and is responsible for half of all deaths of these individuals [1]. Although there are trends toward a fall in this excess mortality risk (perhaps due to earlier detection of type 2 diabetes (T2D) coupled with improvements in care), people with T2D have an elevated risk of CVD compared with those without T2D and a poorer prognosis following an adverse CV event [2,3]. The excess CV risk associated with T2D may arise from a complex interplay of several factors, including chronic hyperglycaemia, hypertension, dyslipidaemia, and obesity [4].

Management of CVD risk factors is therefore of vital importance in T2D care. Improving glycaemic control has provided only limited success in reducing the macrovascular complications associated with T2D [5,6], and so focus has inexorably shifted towards other CV risk factors (including hypertension, dyslipidaemia, and obesity) that contribute to CV morbidity and mortality. More recently, the prevailing assumption that glucose-lowering is inextricably linked with outcome benefit has been challenged [7,8] with the suspension of the European marketing authorisation for rosiglitazone in 2010, and restriction of its use in the U.S., following concerns of an increased risk of CV disorders, including myocardial infarction (MI), heart failure [9], and stroke [10].

**Figure 1:**
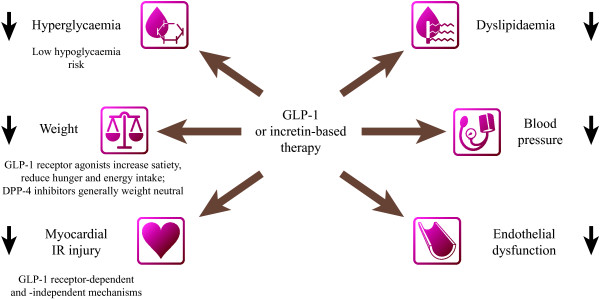
**Mechanisms underlying the putative cardiovascular effects of incretin-based therapies.** ANP, atrial natriuretic peptide, IR, ischaemia-reperfusion.

Determining the optimal therapy regimen for individuals with T2D therefore requires careful consideration, and evidence regarding long-term CV safety of glucose-lowering therapies is required to inform better clinical decision-making. In 2008, the U.S. Food and Drug Administration (FDA) responded to this need by issuing guidelines that mandate a thorough assessment of CV risk in glucose-lowering drug development programmes [11]. The incretin-based therapies are the first drug classes to emerge within this environment of heightened regulatory attention. Clinical studies of incretin-based therapies have demonstrated improved glycaemic control with a low risk of hypoglycaemia and no weight gain, or in the case of glucagon-like peptide-1 (GLP-1) receptor agonists, weight loss [12,13]. These effects, alongside others (summarised in Figure 1) are attractive therapeutic properties in the management of T2D, and signal potential CV benefit. Long-term CV outcomes trials are currently underway for many of the incretin-based therapies (Table 1) [14-24]. This review provides background information on the endogenous incretin system and incretin-based therapies, and examines the available data regarding the effects on CV risk markers and CV safety with incretin-based therapies.

**Table 1:** Randomised clinical trials investigating long-term cardiovascular outcomes with incretin-based therapies in people with T2D

**Title**	**Trial acronym**	**Intervention**	**Enrolment**	**Study duration**	**Primary outcome measure**	**Date initiated (month/year)**	**Primary completion date (month/year)**
**GLP-1 receptor agonists**							
A Randomized Double Blind, Placebo-controlled Clinical Trial to Assess the Effects of Taspoglutide (RO5073031) on Cardiovascular Outcomes in Subjects with Inadequately Controlled Type 2 Diabetes and Established Cardiovascular Disease/NCT01018173	T-EMERGE-8	Taspoglutide 20 mg once weekly	2118	Event-driven timeframe, ≤2 years anticipated	Time to a CV composite endpoint (CV death, acute MI, stroke or hospitalisation for unstable angina)	01/2010	Trial suspended 09/2010 due to high discontinuation rates (gastrointestinal intolerability and serious hypersensitivity reactions)
Liraglutide Effect and Action in Diabetes: Evaluation of Cardiovascular Outcome Results - A Long Term Evaluation/NCT01179048	LEADER	Liraglutide 1.8 mg OD	9340	≤60 months	Time from randomisation to first occurrence of CV death, non-fatal MI or non-fatal stroke	08/2010	01/2016
Exenatide Study of Cardiovascular Event Lowering Trial: A Trial To Evaluate Cardiovascular Outcomes After Treatment With Exenatide Once Weekly In Patients With Type 2 Diabetes Mellitus/NCT01144338	EXSCEL	Exenatide 2 mg once weekly	9500	5.5 years	Time to first confirmed CV event in a composite CV endpoint	06/2010	03/2017
Evaluation of Cardiovascular Outcomes in Patients With Type 2 Diabetes After Acute Coronary Syndrome During Treatment With AVE0010 (Lixisenatide)/NCT01147250	ELIXA	Lixisenatide 20 μg OD	6000	203 weeks	Time to first confirmed CV event	06/2010	09/2014
Researching Cardiovascular Events With a Weekly Incretin in Diabetes/NCT01394952	REWIND	Dulaglutide 1.5 mg once weekly	9622	≤6.5 years	Time from randomisation to first occurrence of CV death, non-fatal MI or non-fatal stroke	07/2011	04/2019
Trial to Evaluate Cardiovascular and Other Long-term Outcomes With Semaglutide in Subjects With Type 2 Diabetes/ NCT01720446	SUSTAIN 6	Semaglutide 0.5 mg or 1.0 mg once weekly	3260	≤148 weeks	Time from randomisation to first occurrence of CV death, non-fatal MI or non-fatal stroke	02/2013	01/2016
**DPP-4 inhibitors**							
Sitagliptin Cardiovascular Outcome Study (0431–082 AM1)/NCT00790205	TECOS	Sitagliptin phosphate 50 mg or 100 mg OD	14000	≤5 years	Time to first confirmed CV event	12/2008	12/2014
Does Saxagliptin Reduce the Risk of Cardiovascular Events When Used Alone or Added to Other Diabetes Medications/NCT01107886	SAVOR- TIMI 53	Saxagliptin 2.5 mg or 5 mg OD	16492	≤2.1 years	Time from randomisation to first occurrence of CV death, non-fatal MI or non-fatal stroke	05/2010	Completed 05/2013
Cardiovascular Outcome Study of Linagliptin Versus Glimepiride in Patients With Type 2 Diabetes/NCT01243424	CAROLINA	Linagliptin 5 mg OD	6000	400 weeks	Time from randomisation to first occurrence of CV death, non-fatal MI, non-fatal stroke or hospitalisation for unstable angina pectoris	10/2010	09/2018
Cardiovascular and Renal Microvascular Outcome Study With Linagliptin in Patients With Type 2 Diabetes Mellitus at High Vascular Risk/NCT01897532	CARMELINA	Linagliptin 5 mg OD	8300	48 months	Time to first occurrence of CV death, non-fatal MI, non-fatal stroke or hospitalisation for unstable angina pectoris	07/2013	01/2018
Cardiovascular Outcomes Study of Alogliptin in Subjects With Type 2 Diabetes and Acute Coronary Syndrome/NCT00968708	EXAMINE	Alogliptin 25 mg OD	5380	40 months	Time from randomisation to first occurrence of CV death, non-fatal MI or non-fatal stroke	09/2009	Completed 06/2013

*Abbreviations*: *CV* cardiovascular, *DPP-4* inhibitors, dipeptidyl peptidase-4 inhibitors, *GLP-1* glucagon-like peptide-1, *MI* myocardial infarction, *OD* once daily.

All are phase 3 clinical studies, except for SAVOR- TIMI 53 and CARMELINA, phase 4 trials. All are placebo-controlled trials, except for CAROLINA (NCT01243424), in which linagliptin 5 mg OD is compared with glimepiride 1–4 mg OD. All studies are double-blinded. Sources: http://www.clinicaltrials.gov; [14-24].

### Evidence acquisition

A literature search of established sources, including congress abstracts, was performed to identify recent publications dealing with the topic for review. Relevant articles were selected for review and discussion in light of the author’s knowledge of the field and clinical judgement. The European label for lixisenatide [25] was consulted for additional information. The official clinical trials registry at http://www.clinicaltrials.gov was also searched to identify long-term CV outcomes clinical trials with incretin-based therapies in individuals with T2D.

### The incretin system and incretin-based therapies

Following nutrient intake, two major incretin hormones, GLP-1 and glucose-dependent insulinotropic polypeptide (GIP), are secreted from the gastrointestinal tract into the circulation [26]. It is now recognised that GLP-1 and GIP are responsible for 50–70% of total insulin secretion after oral glucose intake in healthy individuals [26], augmenting insulin release in a glucose-dependent manner, and leading to reductions in average blood glucose levels. GLP-1 exerts an additional glucose-lowering effect through actions at pancreatic alpha cells which result in an inhibition of glucagon secretion [26]. There is evidence to suggest that the incretin system is functionally impaired in people with T2D, although this is not universally observed [27-31]. This impairment may contribute to the characteristic postprandial hyperglycaemia observed in affected individuals [27]. Native GLP-1 has been shown to reduce hyperglycaemia in T2D; however, as GLP-1 is rapidly (within 2–3 minutes) degraded by the enzyme dipeptidyl peptidase-4 (DPP-4), continuous GLP-1 infusion would be required to maintain optimal glycaemic control [32]. Therefore, incretin-based therapies have been developed either to raise endogenous levels of incretin hormones (DPP-4 agonists) or to mimic GLP-1 effects (GLP-1 receptor agonists) over more extended timeframes.

Pharmacological inhibition of the DPP-4 enzyme with DPP-4 inhibitors increases plasma incretin hormone levels. Several different oral DPP-4 inhibitors are clinically available, including sitagliptin, saxagliptin, linagliptin, alogliptin, vildagliptin (in the EU and Australia), teneligliptin and analagliptin (both available only in Japan) and gemigliptin (available only in Korea). Subcutaneous injection of GLP-1 receptor agonists mimics the effects of native GLP-1 through stimulation of the G_s_-protein coupled GLP-1 receptor. Two GLP-1 receptor agonists are currently widely available: liraglutide (a GLP-1 analogue with 97% homology to native human GLP-1) and exenatide (53% homology to native human GLP-1). Exenatide was introduced to the market in 2007, having been developed from the peptide exendin-4 in the venom of the *Gila* monster, and is administered twice daily (BID). A major advantage of liraglutide when introduced in 2009 was its once daily administration. An extended release once-weekly formulation of exenatide (also known as exenatide long-acting release [LAR]) is also available in both the European Union [33] and in the U.S. [34]. Lixisenatide, a novel GLP-1 receptor agonist, was recently launched in several regions, including the European Union [25], and a number of GLP-1 receptor agonists are currently in clinical development, including albiglutide.

### Cardiovascular effects of native GLP-1

The effects of native GLP-1 on contractility, heart rate and/or blood pressure have been investigated [35-37], but are not well-established. Differing effects of native GLP-1 on these parameters have been observed, presenting a complex picture. These varying effects may arise from differences in species, GLP-1 concentrations and experimental methods used.

Native GLP-1 exerts its effects via the GLP-1 receptor; but may also have GLP-1 receptor-independent effects [38]. The GLP-1 receptor is ubiquitously expressed, located not only in pancreatic cells but also in the lungs, kidneys, intestines, and peripheral and central nervous systems reviewed in [26,37]. In addition, GLP-1 receptors are expressed in the CV system. In mice, GLP-1 receptors are distributed in myocardial tissue (left and right ventricles, septum and, to a lesser extent, atria), endocardium, microvascular endothelium, and coronary smooth muscle cells [38]. GLP-1 receptor expression in the human heart, including the coronary artery endothelial cells, has also been demonstrated [39,40].

Data from animal models and pilot clinical studies have indicated that native GLP-1 may have cardioprotective effects in the setting of ischaemia, or following ischaemic injury [37,38,41,42]. For example, in a study of 21 individuals with acute MI and severe systolic dysfunction after successful primary angioplasty, GLP-1 infusion (in addition to standard care) significantly improved left ventricular function compared with control individuals receiving standard care (including aspirin, clopidogrel, heparin, glycoprotein IIb/IIIa inhibitors, beta-blockers, angiotensin converting enzyme (ACE) inhibitors, and/or statins) [41]. Moreover, studies using the technique of brachial artery flow-mediated vasodilation (measured by ultrasonography) during GLP-1 infusion in people with T2D and stable coronary artery disease suggest that GLP-1 may improve endothelial function in some individuals with T2D [43]. The effects of GLP-1 on endothelial function have been reviewed by Sjöholm [44].

Interestingly, in preclinical studies, cardioprotective effects were apparent in GLP-1 receptor knockout mice, supporting the hypothesis that certain cardioprotective actions of GLP-1 are mediated through GLP-1 receptor-independent pathways [38].

### Effects of incretin-based therapies on cardiovascular risk factors

People with T2D who are overweight or obese, are hypertensive or have dyslipidaemia are at increased risk of adverse CV events. Incretin-based therapies have been shown to have an impact on these CV risk factors; however, differences in these effects between DPP-4 inhibitors and GLP-1 receptor agonists have been noted. These differences may arise from differences in mechanism of action, or levels of GLP-1 receptor activation produced by these individual drug classes [12].

### Weight

Obesity increases the risk of CVD mortality in T2D [45]. As more than half of individuals with T2D are obese [46], weight control is an important aspect of CV risk management in T2D. Encouragingly, lifestyle and certain pharmacological interventions that promote weight loss in people with T2D can improve CV risk profiles [47,48].

DPP-4 inhibitors do not have major effects on weight [12]. Sitagliptin has been associated with changes in body weight of between 0.0 and −1.5 kg [49-52]. Clinical studies examining saxagliptin have shown either dose-independent, numerical decreases (−0.11 to −1.8 kg) or increases (+0.5 to +0.8 kg) in body weight [53-56]. Meanwhile, vildagliptin treatment has been associated with minimal weight gain of 0.2–1.3 kg [57].

In randomized controlled trials, use of the GLP-1 receptor agonists exenatide, liraglutide, or lixisenatide has been associated with body weight reductions. Exenatide BID treatment has been associated with average weight losses of up to 2.8 kg [58-60], and exenatide LAR with mean weight losses of up to 3.7 kg [61-66]. Similarly, in the GetGoal phase 3 development programme, lixisenatide was associated with mean body weight reductions of up to 3.0 kg [67-75], although not all observed weight changes were statistically different from comparators (placebo, exenatide) [67,69,70,75]. In the Liraglutide Effect and Action in Diabetes (LEAD) phase 3 clinical trials, consistent, significant improvements in body weight were observed with liraglutide use versus comparator arms, with average weight losses of up to 3.2 kg [76-81].

Head-to-head trials comparing exenatide LAR [62,64] or liraglutide [82-84] with sitagliptin have confirmed significantly greater changes in weight with the GLP-1 receptor agonists.

### Blood pressure

Hypertension is a risk factor for adverse CV events, raising the risk of coronary heart disease by approximately 2-fold and the risk of stroke by 2- to 4-fold [85,86].

Relatively few studies have examined the effects of DPP-4 inhibitors on blood pressure in people with T2D. Limited evidence suggests that sitagliptin may reduce systolic blood pressure (SBP) [83,87,88], although this effect has not been observed across all studies [89]. Pooled data from six phase 3 clinical trials indicated similar small decreases in SBP and DBP with linagliptin 5 mg once daily and placebo [90].

The clinically available GLP-1 receptor agonists reduce SBP [12,25]. In phase 3, placebo-controlled studies of lixisenatide, reductions of up to 2.1 mmHg were observed [25]. In the case of exenatide BID, reductions in SBP of 3.5 mmHg have been observed, with the greatest improvements seen in those who lost most weight (−8.1 mmHg in 25% of subjects) [91]. Liraglutide treatment was associated with reductions in SBP of up to 6.7 mmHg across the LEAD phase 3 development programme [76-82]. Interestingly, the reductions in SBP observed with liraglutide were observed prior to major weight loss, suggesting that SBP reduction does not occur solely via weight reduction [92]. As would be predicted, meta-analysis of the liraglutide phase 3 trials showed that individuals with the highest baseline SBP (>140 mmHg) exhibited the greatest reductions in SBP (−11.4 mmHg) [93], and that these reductions in SBP were independent of antihypertensive treatment [94].

Weight loss (discussed above), natriuresis and vasodilation [95] are likely to contribute to the SBP-lowering effects of GLP-1 receptor agonists. A recent mouse model study has linked GLP-1 receptor stimulation by liraglutide to cAMP/Epac2-dependent atrial natriuretic peptide release, which would be predicted to increase natriuresis and vasodilation, thereby lowering blood pressure [95]. Consistent with these observations, a GLP-1 receptor-dependent, nitric oxide- and endothelium-independent vasodilatory effect of GLP-1 has been identified in rat femoral artery preparations [96].

### Lipid profiles and other CV risk markers

Elevated fasting triglycerides (TG) and low density lipoprotein cholesterol (LDL-C), and reduced high density lipoprotein cholesterol (HDL-C), are associated with increased risk of CVD [97].

DPP-4 inhibitors have been shown to have either no effects or minor effects on fasting lipid levels in people with T2D [90,98,99]; however, they may have an effect on postprandial lipids. Indeed, significant reductions in postprandial TG, apolipoprotein B-48 (ApoB-48, a LDL), very low density lipoprotein cholesterol (VLDL-C) and free fatty acids (FFAs) have been reported following sitagliptin treatment [postprandial area under the curve (AUC) reduced by 9.4, 7.8, 9.3 and 7.6%, respectively, *p* < 0.05 for all measurements vs. placebo] [100]. Postprandial TG and ApoB-48 reductions and have also been reported following vildagliptin treatment [incremental AUC −2.0 ± 0.8 (*p* = 0.011) and −0.6 ± 0.3 (*p* = 0.008), respectively] [101] and alogliptin treatment [incremental AUC −3.4 ± 0.7 (*p* < 0.001) and −0.9 ± 0.2 (*p* = 0.028), respectively] [102]. The effects of incretin-based therapies on postprandial hyperlipidaemia have been reviewed elsewhere by Ansar and colleagues [103].

While no lipid profile data have yet been published for lixisenatide, meta-analyses have shown overall improvements in fasting lipids with exenatide (BID and LAR) and liraglutide therapy. Liraglutide reduces LDL, FFA and TG levels (by 0.2, 0.09 and 0.2 mmol/L, respectively, *p* < 0.01) and exenatide BID decreases LDL levels (by 0.15 mmol/L, *p* < 0.05 [104]). Trends towards reductions in FFA and TG were also evident with exenatide, but these did not reach statistical significance [104]. Small decreases in HDL reported for both liraglutide and exenatide (0.04 and 0.05 mmol/L, respectively, *p* < 0.01) may not be clinically meaningful. No reductions in VLDL-C were observed for either GLP-1 receptor agonist [104].

Improvements in surrogate CV risk biomarkers, such as high sensitivity C-reactive protein (hsCRP), B-type natriuretic peptide (BNP), and plasminogen activator inhibitor-1 (PAI-1) have also been observed following treatment with liraglutide or exenatide BID [105-108].

### Effects of incretin-based therapies on heart rate and cardiac repolarisation

Substantial resting heart rate elevation is associated with increased CV mortality, and drugs that prolong cardiac repolarisation carry a risk of provoking adverse CV events [109,110]. What are the effects of incretin-based therapies on these parameters?

#### Heart rate

As resting heart rate elevation in excess of 10 beats per minute (bpm) has been positively correlated with CV and all-cause mortality [109], this should be evaluated when considering the CV safety profile of a drug.

A limited amount of data regarding the effects of DPP-4 inhibitors on heart rate is available. Saxagliptin treatment has no apparent effect on heart rate [111]. Following high doses of linagliptin (100 mg), small increases in heart rate (>4 bpm compared with placebo) were observed. However, no meaningful changes in heart rate were observed in individuals who received therapeutic doses of linagliptin (5 mg) in the same study [112].

A randomised, double-blind crossover study of intravenous exenatide infusion (0.12 pmol/kg/min) versus placebo in 20 people with T2D and congestive heart failure identified acute and statistically significant increases in placebo-corrected heart rate of 9, 14 and 15 bpm after 1, 3 and 6 hours of exenatide infusion (0.12 pmol/kg/min), respectively [113]. However, in a separate evaluation of individuals with T2D (n = 54) treated chronically with exenatide BID for 12 weeks, heart rate was not significantly elevated above placebo (least square mean 2.1 ± 1.4 for exenatide vs. -0.7 ± 1.4 bpm for placebo, *p* = 0.16 [114]). Similarly, in one study of healthy volunteers, a transient increase in heart rate was observed with a therapeutic dose of lixisenatide (20 μg), but no mean increases in heart rate were observed with this GLP-1 receptor agonist in phase 3, placebo-controlled studies of individuals with T2D [25]. Studies of exenatide LAR have reported small but significant (*p* < 0.05) increases in heart rate from baseline (least square mean increase of 4 ± 1 bpm [63]) and liraglutide treatment has also been associated with increases in heart rate of 2–4 bpm [76,77,79,81]. The mechanism behind the increase in heart rate observed with certain GLP-1 receptor agonists is not yet well understood. One possible explanation is that this is a compensation for the above-mentioned decrease in SBP.

#### QT interval

The electrocardiographic QT interval is a measure of cardiac repolarisation. An increased QT interval is a risk factor for *torsades de pointes* arrhythmias and sudden cardiac death. Hence, any drug that prolongs the QT interval may increase the risk of adverse CV events reviewed in [110].

The DPP-4 inhibitors sitagliptin [115], saxagliptin [101] and vildagliptin [116] are not associated with QT interval prolongation at clinically relevant concentrations in healthy individuals.

The effects of GLP-1 receptor agonists on QT_c_ interval, that is, corrected for heart rate, have been studied in healthy volunteers. In this population, liraglutide administration (0.6, 1.2. and 1.8 mg OD) produced no significant QT_c_ prolongation [117]. A significant positive correlation between plasma exenatide concentration and QT_c_ interval was reported in one study following single-dose administration of 10 μg exenatide in 62 healthy individuals [regression analysis slope (95% confidence interval; CI), 0.02 (0.01, 0.03), *p* < 0.001; change in QT_c_F (Fridericia correction) interval (90% prediction interval) estimated as 4.95 (2.64, 7.25) ms at geometric mean C_max_ of exenatide] [118]. However, a further QT interval study in which exenatide was administered by intravenous infusion to achieve supratherapeutic plasma concentrations (up to ~630 pg/mL) was reassuring, indicating no QT_c_ prolongation in a population of 74 healthy adults [119]. To date, the effects of lixisenatide on cardiac repolarisation have not been published.

### Potential cardioprotective effects of incretin-based therapies

Consistent with the findings outlined above regarding a cardioprotective effect of native GLP-1 under ischaemic conditions, data from rodent and porcine models indicate that GLP-1 receptor agonists may limit infarct size following ischaemia-reperfusion injury [119-122]. A possible cardioprotective effect of exenatide following myocardial infarction has also been identified in a clinical setting. In people with ST-segment elevation MI undergoing primary percutaneous coronary intervention, intravenous exenatide administration at the time of reperfusion led to a reduction in the area of myocardium at risk, as assessed by cardiac magnetic resonance 3 months after intervention [123]. A *post hoc* analysis tested the hypothesis that this effect would be more pronounced in those with short duration of ischaemia. Data from 148 participants were stratified according to median time from ambulance call or first contact with healthcare system to first balloon inflation (132 min). In subjects with short duration ischaemia (≤132 min), exenatide reduced final infarct size by 30% and increased myocardial salvage index by 14%; in contrast, no such effects were observed in those longer duration ischaemia (>132 min) [124]. Taken together, these findings highlight a potential cardioprotective effect of GLP-1 receptor agonists in the setting of myocardial ischaemia-reperfusion injury, provided they are administered early.

### Long-term effects of incretin-based therapies on cardiovascular disease

Incretin-based therapies were amongst the first T2D treatments for which detailed evaluation of long-term CV safety was encouraged under the 2008 FDA guidance [11]. This guidance recommends that, with regard to the risk ratio for major adverse cardiovascular events (MACE), pre-approval clinical trials should demonstrate that the upper boundary of the two-sided 95% CI is less than 1.8 versus the control group, with subsequent outcomes trials indicating a two-sided 95% CI for risk ratio of less than 1.3 [11].

MACE analyses carried out for sitagliptin [125], saxagliptin [111] and alogliptin [126] have not indicated increased CV risk. Consistent with these findings, a recent meta-analysis identified no increased risk of CV events with a pooled group of DPP-4 inhibitors added to metformin versus metformin monotherapy [relative risk (95% CI) 0.54 (0.25-1.19), *p* = 0.13] [127]. Indeed, DPP-4 monotherapy was associated with a significantly lower risk of CV events than metformin monotherapy [relative risk (95% CI) 0.36 (0.15–0.85), *p* = 0.0.2], indicating a possible CV benefit of DPP-4 inhibitors [127]. Additionally, linagliptin has been associated with significantly fewer CV events than glimepiride over a 2-year period [both in combination with metformin ± one additional oral antidiabetic drug; relative risk (95% CI): 0.46 (0.23–0.91), *p* = 0.0213], despite similar reductions in mean HbA_1c_[128].

MACE analyses conducted for GLP-1 receptor agonists have similarly reported values within the FDA predefined safety limits for diabetes therapies. One meta-analysis of results from randomised trials of GLP-1 receptor agonists (exenatide BID, exenatide LAR, liraglutide, albiglutide) for the period up to November 2010 indicated the following cardiovascular safety margins [Mantel-Haenszel odds ratio for MACE (95% CI)]: all GLP-1 receptor agonists 0.74 (0.50–1.08), *p* = 0.12; exenatide 0.85 (0.50-1.45), *p* = 0.55; liraglutide 0.69 (0.40–1.22), *p* = 0.20 [129]. An as-yet-unpublished meta-analysis of eight, phase 3 studies of lixisenatide indicated a hazard ratio (95% CI) for adjudicated MACE of 1.03 (0.64, 1.66) versus placebo [25]. For liraglutide, retrospective MACE analysis of pooled data from all completed phase 2 and phase 3 randomised trials to date, plus all open-label trial extensions, indicated that the incidence ratio for broad/serious adjudicated MACE versus comparator drugs (metformin, glimepiride, rosiglitazone, insulin glargine) and placebo was 0.73 (95% CI 0.38, 1.41) [130].

These pooled data, mostly from phase 2 and 3 trials, are complemented by reassuring observational data. For example, a retrospective analysis of the U.S. LifeLink™ database of medical and pharmaceutical insurance claims (for the period June 2005 to March 2009) indicated that exenatide BID was not associated with an excess CV risk compared to other glucose-lowering therapies [131]. Consistent with this, another retrospective analysis found no elevated risk of MACE with exenatide BID compared to insulin for ≤1 year [132].

The long-term CV safety and efficacy of many incretin-based therapies are being prospectively evaluated in randomised control trials (Table 1). With the exception of the EXSCEL study, all include people with pre-existing CV disease and/or people with high risk for CV disease [16-24]. As of September 2013, a body of evidence is beginning to emerge on the long-term CV safety profile of incretin-based therapies in individuals at high risk of CV events: the results of the first two trials (SAVOR-TIMI 53 [saxagliptin] [22] and EXAMINE (alogliptin [24]), published as this article went to press, meet the FDA criteria for non-inferiority of these agents over placebo but provide no positive evidence of CV risk reduction. More evidence is eagerly awaited to inform clinical decision-making when seeking to minimise the risk of CVD in people with T2D.

## Conclusions

In addition to their glucose-lowering properties, incretin-based therapies have apparently beneficial effects on CV risk factors, accompanied by small effects on heart rate, without clinically meaningful QT_c_ prolongation. There is mechanistic evidence to suggest that GLP-1 receptor agonists may confer cardioprotective effects following ischaemia. Retrospective MACE analyses conducted for the different incretin based therapies have been reassuring. However, as mandated by regulatory agencies, the long-term CV safety of DPP-4 inhibitors and GLP-1 receptor agonists is currently under investigation in large CV outcomes trials. The results – which will emerge over the next few years – may also provide data on the efficacy of incretin-based agents in preventing CV events. This information will supply the evidence needed to position the use of these classes rationally amongst other more established glucose-lowering therapies.

## Abbreviations

ApoB-48: Apolipoprotein B-48; ACE: Angiotensin converting enzyme; AUC: Area under the curve; BID: Twice daily; BNP: B-type natriuretic peptide; bpm: Beats per minute; CI: Confidence interval; CV: Cardiovascular; CVD: Cardiovascular disease; DPP-4: Dipeptidyl peptidase-4; FDA: U.S. Food and Drug Administration; FFA: Free fatty acid; GIP: Glucose-dependent insulinotropic polypeptide; GLP-1: Glucagon-like peptide-1; HDL-C: High density lipoprotein cholesterol; hsCRP: High sensitivity C-reactive protein; LAR: Long-acting release; LEAD: Liraglutide effect and action in diabetes; LDL-C: Low density lipoprotein cholesterol; MACE: Major adverse cardiovascular events; MI: Myocardial infarction; OD: Once daily; PAI-1: Plasminogen activator inhibitor-1; QTc: Heart rate-corrected QT interval; QTcF: Fridericia correction QT interval; SBP: Systolic blood pressure; T2D: Type 2 diabetes mellitus; TG: Triglyceride; VLDL-C: Very low density lipoprotein cholesterol.

## Competing interests

JP has received honoraria for lectures, travel support and consultancy services from pharmaceutical companies manufacturing diabetes treatments, including AstraZeneca, Bristol Myers Squibb, GlaxoSmithKline, Novo Nordisk, Roche/Genentech, Sanofi-Aventis and Takeda. He serves on GLP-1 agonist clinical trial committees for Novo-Nordisk and Sanofi-Aventis. He is a recipient of support in kind from Merck-Serono for a JDRF-funded investigator-led study (REMOVAL, NCT01483560; http://clinicaltrials.gov/show/NCT01483560).
